# Detection and Analysis of C-to-U RNA Editing in Rice Mitochondria-Encoded ORFs

**DOI:** 10.3390/plants9101277

**Published:** 2020-09-28

**Authors:** Peng Zheng, Dongxin Wang, Yuqing Huang, Hao Chen, Hao Du, Jumin Tu

**Affiliations:** 1 Zhejiang Provincial Key Laboratory of Crop Germplasm Resources, Institute of Crop Science, Zhejiang University, No. 866, Yu-Hang-Tang Road, Hangzhou 310058, China; 11716009@zju.edu.cn (P.Z.); isco@zju.edu.cn (Y.H.); haochen@zju.edu.cn (H.C.); 2 College of Life Science and Technology, Guangxi University, Nanning 530004, China; biowangdongxin@163.com

**Keywords:** C-to-U RNA editing, mitochondria-encoded ORFs, edited codon, rice

## Abstract

Cytidine to uridine (C-to-U) RNA editing is an important type of substitutional RNA modification and is almost omnipresent in plant chloroplasts and mitochondria. In rice mitochondria, 491 C-to-U editing sites have been identified previously, and case studies have elucidated the function of several C-to-U editing sites in rice, but the functional consequence of most C-to-U alterations needs to be investigated further. Here, by means of Sanger sequencing and publicly available RNA-seq data, we identified a total of 569 C-to-U editing sites in rice mitochondria-encoded open reading frames (ORFs), 85.41% of these editing sites were observed on the first or the second base of a codon, resulting in the alteration of encoded amino acid. Moreover, we found some novel editing sites and several inaccurately annotated sites which may be functionally important, based on the highly conserved amino acids encoded by these edited codons. Finally, we annotated all 569 C-to-U RNA editing sites in their biological context. More precise information about C-to-U editing sites in rice mitochondria-encoded ORFs will facilitate our investigation on the function of C-to-U editing events in rice and also provide a valid benchmark from rice for the analysis of mitochondria C-to-U editing in other plant species.

## 1. Introduction

RNA editing is an essential post-transcriptional biological process that specifically changes the nucleotide sequence of a primary transcript, making the genetic information in RNA different from that of the DNA template. The term RNA editing was introduced for the first time in 1986 to describe the addition and deletion of uridine nucleotides to and from mRNAs in the kinetoplast, the specialized mitochondrion of trypanosomes [1]. Since its initial discovery, RNA editing has been found in a wide range of organisms, including basal eukaryotes, land plants, vertebrates, fungi, and viruses as well [2]. RNA modifications due to RNA editing comprise nucleotide insertions/deletions or substitutions that can occur in the nucleus, in the cytoplasm, as well as in organelles (plastids and mitochondria) [3].

RNA editing in plant organelles occurs mainly in the form of cytidine to uridine (C-to-U) conversion, although other types of RNA editing including the opposite U-to-C alteration has also been seen especially in chloroplasts RNAs of some taxa of land plants [4]. In flowering plants, several hundred C-to-U substitutions are observed in the two energy-producing organelles, with the majority located in mitochondria. Plant C-to-U editing was found to be associated with nucleus-encoded pentatricopeptide repeat (PPR) proteins [5], which are characterized by 31–36 amino acid repeated motifs that can recognize and bind single-strand RNAs [6]. Several studies have demonstrated that other non-PPR proteins, including multiple organellar RNA editing factor (MORF) [7] and organelle RNA recognition motif-containing (ORRM) proteins [8,9], are required to form the editosome machinery in flowering plants, although its molecular assembly mechanism remains largely unknown [10].

Most plant organellar C-to-U RNA editing occurs in protein-coding regions (usually at the first or second position of codons), typically leading to amino acid changes that appear to be evolutionarily conserved [11]. Although C-to-U alteration most frequently modifies internal codons, in rare cases it also creates translation initiation (ACG-to-AUG) or termination codons (CAA-to-UAA). Therefore, this RNA modification is believed to act as a proofreading mechanism to correct DNA mutations at the RNA level and to generate functional proteins [12]. C-to-U RNA editing occasionally occurs in untranslated regions (5′ UTR or 3′ UTR), introns, rRNA and tRNA molecules, modulating splicing, transcript stability, and translation efficiency [3]. Indeed, several RNA editing events in introns have proved to improve the stability of functionally relevant secondary structure motifs [13].

With the release of many complete plant organellar genomes, RNA editing events in chloroplasts and mitochondria of many plant species have been identified through direct Sanger sequencing [14,15,16] or high-throughput sequencing of transcriptomes [17,18,19,20]. C-to-U RNA editing sites can also be detected by specifically developed prediction tools, such as PREPACT [21], CURE-Chloroplast [22], and the PREP suite [23].

Several studies have demonstrated that C-to-U RNA editing is essential for normal plant growth and development. The alteration of C-to-U RNA editing pattern can lead to a series of plant developmental defects, such as impaired chloroplast and mitochondrial biogenesis [24], retarded seedling growth [25], reduced embryo and endosperm development [26], and hypersensitivity to various abiotic stresses [27]. Moreover, loss of C-to-U RNA editing in plant mitochondria can result in male sterility, also termed as cytoplasmic male sterility (CMS) [28]. These findings suggest that C-to-U RNA editing plays important roles in various plant developmental processes, including organelle biogenesis, adaptation to environmental changes, and signal transduction.

Rice is not only a major staple food crop for more than half of the population worldwide, it is also an important monocot model for basic molecular and genetic studies. Detection of C-to-U RNA editing sites in rice mitochondria will not only facilitate the functional investigation of genes essential for mitochondria biogenesis and plant development, but will also provide valuable clues to generate CMS lines to improve hybrid rice production. A previous study has identified a total of 491 C-to-U RNA editing sites in 33 open reading frames and one pseudogene of the rice mitochondrial genome [29]. Editing sites are not always correctly or completely annotated in primary databases such as GenBank, where they are often indicated as mis-features or as simple exception notes. To overcome these limitations, researchers have developed a series of specialized databases, among which REDIdb was the first specialized database to collect plant RNA editing events and annotate them in their biological context [30]. The latest version of REDIdb contains 26,618 RNA editing sites distributed among 281 organisms, including those C-to-U RNA editing sites in rice mitochondria. The availability of well-annotated mitochondrial editing sites through specialized databases has been beneficial for identifying the biological functions of several C-to-U RNA editing events in rice mitochondria [31,32,33]. Nevertheless, missing or inaccurately annotated editing sites have been observed by independent research groups. These problems, as a consequence, will hamper and even preclude the functional identification of rice genes involved in C-to-U editing or containing C-to-U editing sites.

With the aim of finding novel C-to-U RNA editing sites and correct potential annotation errors, we first used direct Sanger sequencing of cDNAs amplified by RT-PCR to verify rice mitochondria-encoded ORFs [29], including 35 known genes, two pseudogenes, and 10 ORFs with unknown functions. To prove the authenticity of these editing sites, RNA-seq data retrieved from the public database were then exploited for confirmation. In total, we identified 569 C-to-U RNA editing sites in rice mitochondria, 78 more than the number reported previously [29]. Total 85.41% of these editing sites were observed on the first or the second base of a codon, resulting in alteration of the corresponding encoded amino acid. We also found some novel editing sites and several inaccurately annotated sites. Finally, we comprehensively annotated all 569 C-to-U RNA editing sites. More precise information about C-to-U RNA editing sites in this study will facilitate our investigation on the function of C-to-U editing events in rice, and at the same time provide a valid benchmark from rice for the analysis of mitochondria C-to-U editing in other plant species.

## 2. Results

### 2.1. C-to-U RNA Editing Sites Detection in ORFs of Rice Mitochondrial Genome

Given the fact that most editing sites locate in the protein-coding regions, we only detected C-to-U modifications in rice mitochondria-encoded ORFs. By analyzing gene organization in the rice mitochondrial genome, Notsu et al. (2002) identified 35 genes for annotated proteins, two pseudogenes for ribosomal proteins, and several genes for tRNA and rRNA [29]. By comparing the mitochondrial cDNA sequences with the mitochondrial genome sequence, a total of 491 C-to-U editing sites were detected in the 33 protein-coding genes and one pseudogene in rice mitochondria (Table 1). In this study, in addition to the 35 ORFs for known proteins and two pseudogenes, we also investigated the 10 ORFs (*orf152a*, *orf152b*, *orf161*, *orf153*, *orf162*, *orf165*, *orf176*, *orf187*, *orf224*, and *orf288*), which are capable of encoding more than 150 amino acids and have been proved to be transcribed, even though no RNA editing site was found in any of these 10 ORFs in a previous study [29].

C-to-U editing sites in the rice mitochondria-encoded ORFs were first detected in rice leaves by Sanger sequencing. In total we identified 569 significant C-to-U editing sites defined as having a threshold editing level above 10% (Supplementary Materials Table S1). Considering that both potential cloning artefacts and sequencing artefacts can lead to false positives, an alternative approach based on RNA-seq was used to check whether edits identified by Sanger sequencing were genuine editing sites or not. To avoid tissue-specific editing events, RNA-seq data from leaf samples were selected for analysis. In total, 205,435,765 short reads were obtained by sequencing cDNA obtained from two leaf samples. We aligned these reads to rice mitochondrial targets, recovering 1,010,422 unique alignments. The result showed that 567 out of the 569 editing sites were again identified as sites with editing levels of 10% or greater. The editing levels of other two sites, *nad3*-154 and *orfB*-219, were around 9%, slightly lower than the threshold 10% by RNA-seq while more than 10% by Sanger sequencing (Figure S1, Table S2), hence these two sites were still regarded as editing sites. Moreover, through RNA-seq analysis, we identified another 13 editing sites distributed in different ORFs, whose editing levels were slightly higher than 10% (Table S2). However, 11 out of the 13 sites were located in the third base of the codons, resulting in synonymous amino acid changes. Furthermore, editing of these sites were not detected by Sanger sequencing, thus these 13 sites were not considered as editing sites. For reference, the coverage of each edited positions, which is from 74 reads for *matR*-17 to 9487 reads for *cox3*-572, is listed in Table S2. High congruence of the editing levels for each site was observed when RNA-seq data from two leaf samples were analyzed separately (Table S2), suggesting a high reliability of C-to-U editing sites detected in this study.

All these 569 RNA modifications were distributed in 37 different ORFs (Table 1). Compared with the data from Notsu et al. (2002), although there were no or few differences in the number of editing sites in most genes, notable differences were observed in several genes. For example, one editing site in *cox3* and four in *orfB* were identified by Notsu et al. (2002), while in this study 13 and seven editing sites were detected in *cox3* and *orfB*, respectively. Interestingly, we also identified 16 C-to-U editing sites in *matR,* and 6 sites in *rps12* (Table 1), but no C-to-U substitution has been reported in these two genes previously. Among those ten ORFs capable of encoding more than 150 amino acids, only three sites in *orf288* (*orf288*-56, -58, *and* -134) were edited with low editing level as revealed by Sanger sequencing and RNA-seq data (Tables S1 and S2). The numbers of C-to-U editing sites in each transcript are documented in Table 1. For reference, we have also compiled in Table 1 the numbers of C-to-U editing sites annotated in REDIdb [30].

### 2.2. Correction of Annotation Errors in REDIdb

**Figure 1:**
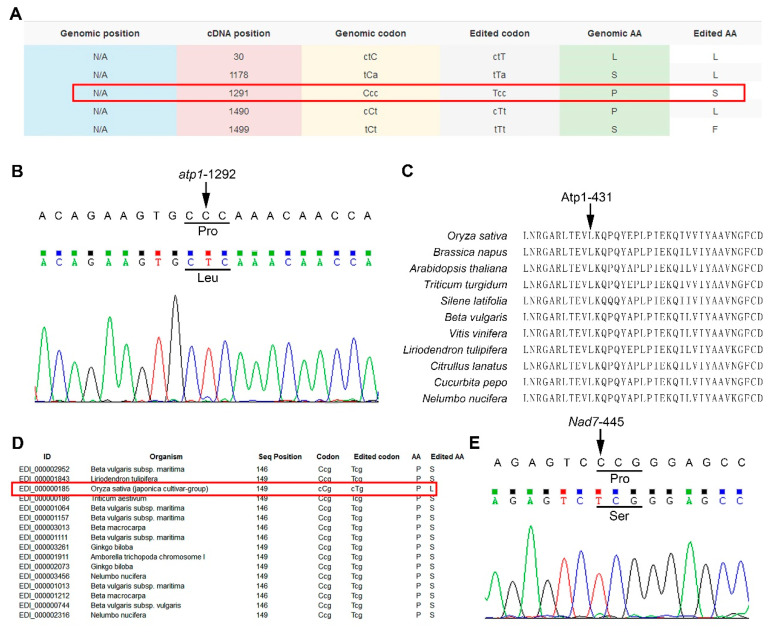
Confirming and correcting the annotations in REDIdb. (**A**) Annotation of RNA editing sites in *atp1* transcript in REDIdb. Red box indicates the incorrectly annotated RNA editing site at *atp1*-1291. (**B**) Sequence chromatogram of PCR-amplified *atp1* cDNA. Arrow indicates the editing site *atp1-*1292. Amino acids before and after editing are shown below the codons indicated by black lines. (**C**) Alignment of amino acid sequences of rice Atp1 and its orthologs from other plants species around the amino acid Atp1-431. The sequences were retrieved from REDIdb and aligned with Clustal Omega (http://www.ebi.ac.uk/Tools/msa/clustalo/) [34]. Arrow indicates the amino acid for rice Atp1-431 encoded by the edited codon. Note that amino acid sequences from REDIdb are translated from edited transcripts. (**D**) Annotations retrieved from REDIdb on the amino acid alteration at Nad7-149 in rice and its counterpart in other species. Red box indicates the alteration of amino acid 149 resulting from incorrectly annotated RNA editing site of rice *nad7*-446. (**E**) Sequence chromatogram of PCR-amplified *nad7* cDNA. Arrow indicates the editing site *nad7-*445. Amino acids before and after editing are shown below the codons indicated by black line.

REDIdb is a unique database focusing on collecting and annotating plant organellar RNA editing sites [30]. In its third and most recent release, all annotations were manually checked to correct potential errors. This does not mean, however, that there are no annotation errors still existing in the bioinformatics resource. For example, in accession AB076666 (the new accession for Nipponbare mitgenome is BA000029.3), the annotation for a C-to-U editing in rice mitochondrial gene *atp1* is reported at nucleotide 1291, which results in the conversion of amino acid from Pro to Ser (Figure 1A). On the basis of sequencing results from three biological replicates, we found that the actual editing site is located one nucleotide downstream of the reported editing site at 1292, where a modification at the second codon position converts a genetically encoded amino acid Pro into Leu (Figure 1B). This is also well-confirmed by our multiple alignment result indicating that the Leu at that position is evolutionarily conserved in other plants (Figure 1C). Coincidentally, a C-to-U substitution in the *nad7* transcript is annotated as being located at nucleotide 446, where the residue Pro encoded by CCG codon is converted to Leu encoded by CUG (Figure 1D). However, our sequencing result indicates that the bona fide C-to-U editing site occurred at nucleotide 445 (Figure 1E), creating a UCG codon to encode a conserved Ser as revealed in the annotation (Figure 1D). To improve the annotation quality and facilitate further study of C-to-U editing sites in rice mitochondria, we have corrected all the annotation errors in REDIdb and compiled them in Table S1.

### 2.3. Characterization of Editing Sites in Rice Mitochondria

All 569 C-to-U RNA editing sites in rice mitochondrial genome are unevenly distributed among different genes, with the percentage of editing events per gene C content ranging from 0.29% (*rpl2*) to 23.18% (*ccmB*) (Table S3). No significant correlation was observed between edited Cs and total Cs (*r* = 0.291, *p* < 0.081) or gene length (*r* = 0.259, *p* < 0.121) by means of Pearson’s correlation (Table S2). The *ccmFN* gene, encoding N-terminal maturation subunit F of cytochromes *c*, is the most edited gene (38), whereas the *rpl2* and *rpl5*, both encoding ribosomal proteins, are the least edited genes (1 for each) (Table 1). The number of editing sites for individual genes of a given mitochondrial complex is also quite variable. Only 11 C-to-U RNA editing events were identified in *nad4L* gene, while 34 editing sites were found in *nad7* (Table 1). Our data also confirm a level of species specificity of C-to-U editing. For instance, five edited sites were detected in the rice *cox1* transcript, whereas the orthologs from *Arabidopsis thaliana* and *Vitis vinifera* have 0 and 22 edits, respectively [30,35].

**Figure 2:**
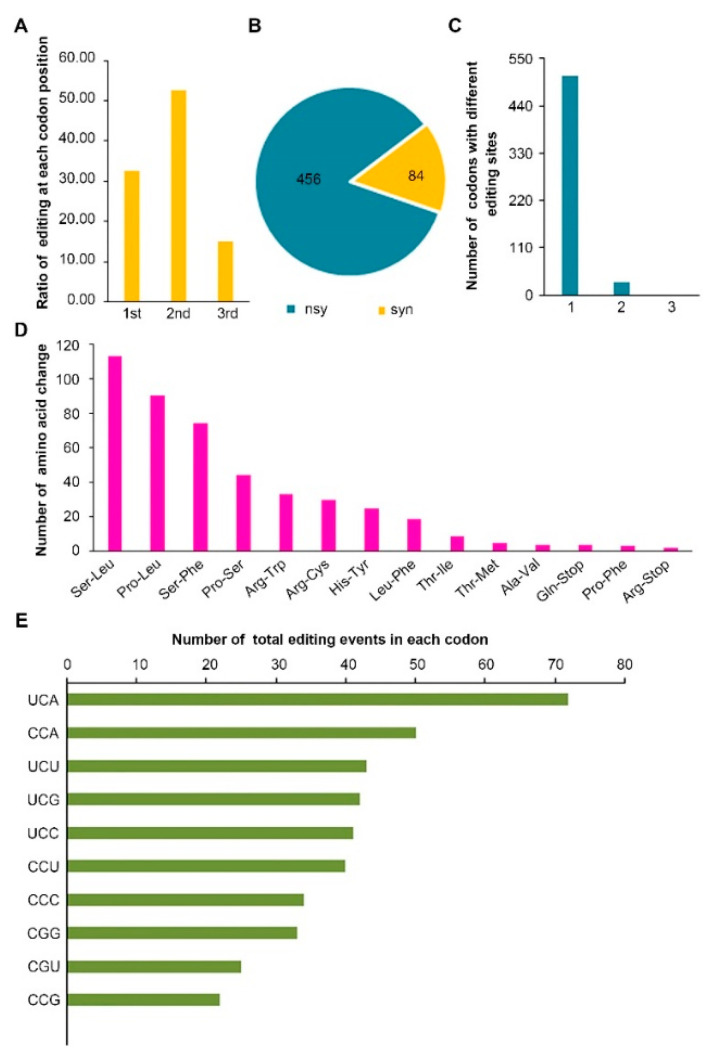
Principal statistics of C-to-U RNA editing sites in ORFs of rice mitochondria. (**A**) Distribution of editing sites at each codon position. (**B**) Number of nonsynonymous (nsy) and synonymous (syn) amino acid substitutions by RNA editing. (**C**) Number of codons with different number of editing sites. (**D**) Number of amino acid changes induced by C-to-U RNA editing. (**E**) Distribution of editing events in each codon.

Among 569 C-to-U RNA editing sites detected in this study, 32.51% (185) and 52.90% (301) occurred at the first and second base of a codon, respectively, and only 14.59% (83) occurred at the third base of the codon (Figure 2A). This frequency is similar to that observed in mitochondria of *V. vinifera* [35]. All the C-to-U modifications resulted in 540 codon alterations, of which 84 were synonymous (AA is unchanged) and 456 were nonsynonymous (AA is changed) (Figure 2B). The number of editing sites per codon was not limited to one. Actually, 511 out of the 540 codons harbor only a single editing site, and the rest 29 codons possess two editing sites for each. No codon that contains three editing sites was detected (Figure 2C). The three most frequent nonsynonymous changes induced by C-to-U editing were Ser-to-Leu (113), Pro-to-Leu (90), and Ser-to-Phe (75) (Figure 2D). These three changes from hydrophilic to hydrophobic lead to the increase of content of hydrophobic amino acids. Indeed, 83.55% of the nonsynonymous changes in the rice mitochondria eventually produced hydrophobic amino acids, suggesting a critical biochemical function for the proteins encoded by the edited transcripts. In plants, these codon conversions mostly restore the expression of evolutionarily conserved amino acid. Moreover, Ser-to-Leu and Ser-to-Phe conversions potentially increase the hydrophobic residues in protein–protein interface while Pro-to-Leu substitutions can contribute to protein function by stabilizing 3D structures [36].

As mentioned above, C-to-U editing sites at 539 codons in rice mitochondria are characterized by non-random distribution with regard to codon positions. Additionally, the bias of editing events toward specific codons were also observed. In particular, the top three edited codons were UCA (73) followed by CCA (50) and UCU (43), accounting for 30.74% of all edited codons (Figure 2E). GCC and AGC are the only two C-containing codons that were never affected by C-to-U alteration. RNA editing in rice mitochondria creates the site of translation initiation in *nad1* and *nad4L* transcripts, and induces six stop codons in *atp6*, *atp9*, *ccmFC*, *rpl16*, *rps1*, and pseudo-*rps11* genes (Table S1).

### 2.4. Novel C-To-U Editing Sites May Be Functionally Important

Besides the discovery of incorrectly annotated C-to-U events, we detected 78 novel editing sites. Some of these sites with low editing levels induced synonymous editing, whereas some sites highly edited generally resulted in nonsynonymous modification. Since the important biological function of several highly edited C-to-U editing sites located in the transcripts has been elucidated, it is possible that these highly edited sites are also functionally important.

**Figure 3:**
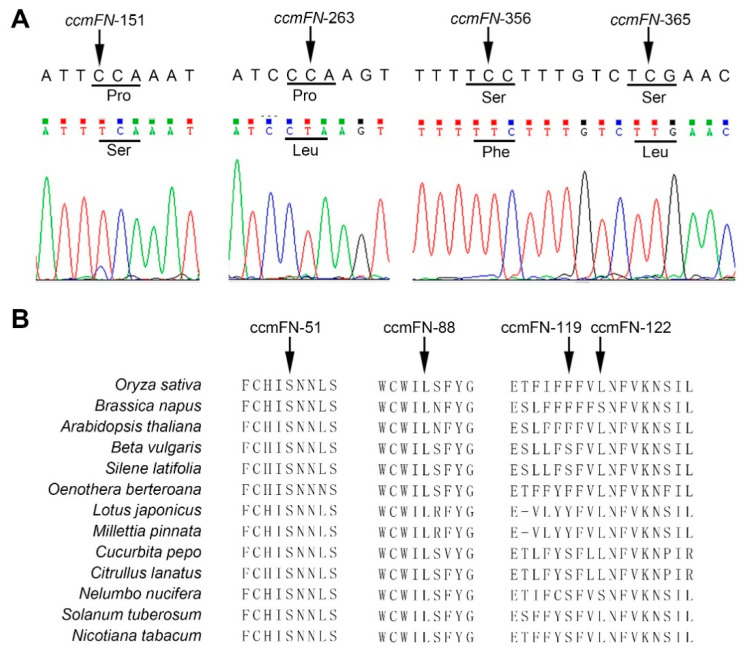
Identification of novel RNA editing sites in rice mitochondria-encoded ORFs. (**A**) Sequence chromatogram of PCR-amplified *ccmFN* cDNA. Arrow indicates the novel editing sites *ccmFN-*151, *ccmFN-*263, *ccmFN-*356, and *ccmFN-*365. Amino acids before and after editing are shown below the codons indicated by black lines. (**B**) Alignment of amino acid sequences from rice CcmFN and its orthologs from other plant species. The sequences of *Oryza sativa*, *Citrullus lanatus*, *Lotus japonicus*, *Cucurbita pepo*, *Oenothera berteroana*, *Arabidopsis thaliana*, *Silene latifolia*, *Silene latifolia*, *Nelumbo nucifera* and *Millettia pinnata* were retrieved from REDIdb and sequences of *Nicotiana tabacum* (BAD83458.2), *Beta vulgaris* (NP_063987.2) and *Solanum tuberosum* (QEQ76363.1) were downloaded from NCBI. All the sequences were aligned with Clustal Omega (http://www.ebi.ac.uk/Tools/msa/clustalo/) [34]. Arrows indicate the amino acids for rice CcmFN-51, CcmFN-88, CcmFN-119, and CcmFN-122 encoded by edited codons. Note that amino acid sequences from NCBI are translated from unedited transcripts.

For instance, *ccmFN* encodes a subunit of the heme lyase complex required for cytochrome *c* maturation. In maize, RNA editing of the *ccmFN* transcript at position 1553 results in amino acid conversion from Ser to Phe. Abolishment of C-to-U editing at this site leads to the loss of CcmFN protein and a marked reduction of *c*-type cytochrome, and eventually compromises the development of the maize embryo and endosperm [37]. Actually, C-to-U editing-induced Phe at this site is highly conserved across different species, indicating that nonsynonymous C-to-U modification at some positions, at least at position 1553, is essential for the function of CcmFN protein. In this study, we observed four new highly edited sites in *ccmFN* transcript (Figure 3A). Editing at positions 121, 263, 356, and 365 induced Pro-to-Ser, Pro-to-Leu, Ser-to-Phe, and Ser-to-Leu nonsynonymous substitutions, respectively (Figure 3A). Multiple alignment of CcmFN proteins from various plant species indicated that these four amino acids encoded by the edited codons are highly conserved (Figure 3B). Likewise, five novel sites with high editing levels were detected at the 3′ end of the coding region of *matR* gene, namely *matR*-1745, *matR*-1766, *matR*-1892, *matR*-1900, and *matR*-1910 (Figure S2A). Editing at these sites resulted in nonsynonymous amino acid substitutions (Figure S2A), which are highly conserved among different species as indicated by the result of multiple alignment (Figure S2B). Compared with synonymous editing sites, nonsynonymous editing sites commonly showed higher conservation levels as well as editing levels [30,38], and nonsynonymous sites are more essential and functional, suggesting that detection of novel C-to-U editing sites in this study will provide valuable source for rice biological study.

## 3. Discussion

In plants, RNA editing was first identified as C-to-U conversions in mitochondrial mRNA for *cox2* [39], followed by its identification in chloroplast mRNA for *rpl2* [40]. Since then, the occurrence of C-to-U editing has been widely discovered in plant organelles. With the advent of next-generation sequencing (NGS) technique, numerous novel RNA editing events have been uncovered based on the release of many complete plant organellar genomes and RNA-seq data. Databases have also been developed to collect and annotate the greatly increased C-to-U RNA editing sites [30,41,42]. However, the existence of annotation errors and the lack of novel editing sites in the database will, to some extent, limit the study uncovering the biological functions of particular C-to-U editing events.

Sanger sequencing is the conventional strategy to identify RNA editing sites by direct comparison of cDNA sequences with their corresponding genomic templates. Despite its time-consuming and costly nature, Sanger sequencing is still a suitable and popular method for verifying a relatively small number of editing events [43,44]. In recent years, high-throughput RNA-seq approach by NGS has been used to systematically identify RNA editing sites in different species. NGS technique has many advantages over Sanger sequencing in detecting editing events, one of which is that NGS allows a fast and genome-wide identification of RNA editing sites [17,44]. In spite of this, it is still impossible to find the exact number of all editing sites in plants by NGS technique because of the existence of tissue-specific editing events in different tissues. Moreover, the number of editing sites identified by RNA-seq is also based on the settings for mapping the RNA-seq reads to genomic sequences: relaxed settings may lead to false positives, while more stringent mapping settings may increase the number of false negatives [45]. In this study, 569 C-to-U editing sites in the rice mitochondria-encoded ORFs were first detected by Sanger sequencing, and subsequently most of these editing sites were further confirmed by RNA-seq data. These results indicate that all the editing sites found in the rice mitochondria-encoded ORFs are reliable. Compared with 491 C-to-U editing sites previously identified in rice mitochondria by Sanger sequencing, we detected about 80 novel editing sites. Major discrepancies were observed in three ORFs, with 13, 16, and 6 editing sites detected in this study while only one, zero, and zero detected previously in *cox3*, *matR*, and *rps12*, respectively [29]. The difference in the number of editing sites obtained in these two studies is partial because of the discrepant analysis methods. In the previous study, only the nucleotide indicated by the higher peak in the sequence chromatogram was adopted for analysis, which will inevitably miss some real but low-edited sites (below 50%) [29]. While in this study, both T and C represented by two overlapped peaks in the same position were used to determine the editing sites, and editing levels no less than 10% were identified as real edits.

However, in this study only leaves were used as samples to detect C-to-U editing sites, which consequently discarded the sites specifically occurring in other rice tissues, although only a limited fraction of edited sites in mitochondria have shown to be tissue specific [35]. Moreover, in both methods, the sites with editing level no less than 10% were considered as C-to-U editing sites. These setting parameters will inevitably ignore some genuine editing sites that are edited at extremely low levels, given the fact that editing levels of some sites are very dynamic during plant development and growth [43,46,47]. Hence, it is possible that more editing sites in rice mitochondrial ORFs, and some tissue-specific editing sites will be found if more different rice tissues are used.

Transfers of organelle DNA to the nucleus is a ubiquitous and ongoing process among plants, resulting in the so-called nuclear plastid (NUPT) and nuclear mitochondria (NUMT) [48,49]. In rice, thirty-eight percent of the mitochondrial DNA segments have been inserted into nuclear genome [49], and these nuclear-integrated DNA segments show high similarity to their mitochondria counterparts [50]. Theoretically, the transcripts encoded by NUMTs will affect the identification of C-to-U editing sites by means of non-specific PCR amplification in Sanger sequencing method or incorrect calling of reads in RNA-seq method. However, in order to acquire the function in nucleus, a few single genes in NUMTs usually need to acquire the appropriate controlling elements, such as promoters [51]. But often, the transferred DNA segments were not functional [52]. Recently, some studies showed that different modifications, including DNA methylation, histone tail modifications, and mutation patterns, play essential roles in suppressing the transcription of integrated organelle DNAs [53,54]. Almost all present-day NUMTs give rise to non-coding sequences or pseudogenes [48,50,55], which was supported by the purifying selection evidence [52]. Therefore, the presence of NUMTs in rice cannot cause substantial impact on the identification of C-to-U editing sites in this study.

Although the exact functions of most mitochondrial RNA editing events are unclear, some studies of rice PPR mutants that abolish C-to-U conversions at particular sites may prove the biological significance of these editing events. For example, the complete loss of seven RNA modifications on five mitochondrial transcripts in *ogr1* mutant results in delayed seed germination, retarded growth, and sterility [33]. In *pps1* RNAi plants, significant reduction of editing efficiency at five consecutive editing sites in *nad3* decreases the activity of several complexes in mitochondrial electron transport chain, resulting in delayed development and partial pollen sterility [31]. The loss of C-to-U RNA editing in these mutants and the associated development defect suggest that RNA editing to restore evolutionarily conserved amino acids in rice mitochondrial transcripts may be important for the function of encoded proteins. In this study, we discovered numerous novel editing sites and several incorrectly annotated sites via RT-PCR and Sanger sequencing. Some of these are highly edited and result in the restoration of conserved amino acids, implying that C-to-U RNA editing at these sites may have important biological functions. This study has provided a more accurate and comprehensive information about C-to-U RNA editing sites in rice mitochondria, which will not only offer an excellent opportunity to investigate the effect of RNA editing on mitochondrial function, but also facilitate the functional identification of rice genes involved in C-to-U RNA editing events. Considering that most mitochondrial C-to-U editing sites are highly conserved among different plant species, this study also provides a reference for study of RNA editing sites in other plants.

## 4. Materials and Methods

### 4.1. RNA Isolation and cDNA Synthesis

The complete mitochondrial genome sequence and information on the RNA editing sites in rice mitochondria were previously determined in *japonica* cultivar Nipponbare [29]. To keep the genetic background the same, rice leaves used for detection of C-to-U RNA editing sites were harvested from Nipponbare.

cDNAs used for Sanger sequencing were amplified by reverse-transcription polymerase chain reaction (RT-PCR). For RNA isolation, rice seedlings were cultured in Yoshida culture solution in a growth chamber with a 13-h-light (28 °C)/11-h-dark (28 °C) photoperiod, 50% to 60% humidity, and 6800 to 7000 lx light intensity. Two-week old rice leaves were frozen in liquid nitrogen, ground in a mortar with pestle, and extracted with RNAiso Plus reagent (TaKaRa, Shiga, Japan). Quality and concentration of total RNA were analyzed using a NanoDrop 1000 spectrophotometer (Thermo Fisher Scientific, Waltham, MA, USA). The first strand cDNA was synthesized with PrimeScript RT reagent Kit with gDNA eraser (TaKaRa, Shiga, Japan) following the manufacturer’s protocol. To ensure that the whole CDS regions can be covered by Sanger sequencing, forward primers used for PCR and sequencing were about 40 bp upstream of the initial codons while reverse primers used for PCR were behind the stop codons. Total of 2 µL of a 10-fold dilution of the cDNA solution were used as a template for RT-PCR. PCR products were purified with an AxyPrepTM PCR Cleanup kit (AXYGEN, Union City, CA, USA) and subjected to sequencing using ABI PRISM 3700. All primers for RT-PCR are listed in Table S4.

### 4.2. Identification of RNA Editing Sites by Sanger Sequencing

RNA editing sites were identified in two ways: 1, cDNA sequences were aligned with their corresponding genomic sequences in BLASTN to obtain the mismatched sites. If the unpaired bases were T in cDNA but C in the genomic DNA, this site was considered as a C-to-U editing site. 2, Sequencing chromatograms were directly read to find the double-peaked sites. In such cases, if the two peaks indicate C and T, while a C is observed at the same position in the reference genome, the site was regarded as an editing site or not based on the editing level that was calculated with T/(C + T) formula, the values of C and T were determined by the relative height of C and T peaks in the sequence chromatogram. The sequencing quality indicated by sequence peaks is dependent on the DNA sequence distribution and structure of PCR products as well as quality of primers and purified PCR products. To avoid the false-positive variants caused by the above-mentioned technical limitations, double-peaked sites with editing levels above 10% in at least two of the three biological replicates were regarded as RNA editing sites (extremely low level is possibly caused by sequencing errors). Editing extents of individual sites were categorized as extremely high (EH), high (H), middle (M), or low (L) based on the calculated editing levels of above 90%, 60–90%, 30–60%, and 10–30%, respectively. Known C-to-U substitutions in rice mitochondria were downloaded from the REDIdb database and served as reference to identify new editing sites. Two editing sites in a single codon were annotated together.

### 4.3. Identification of RNA Editing Sites from RNA-Seq Data

We designed a custom computational pipeline to identify editing sites by using two inputs: a reference file with the rice mitochondrial ORFs and a file with RNA-seq data. The reference file existed in a multi-FASTA file containing the CDS of ORFs. These sequences were extracted from complete mitochondrial genome available in the GenBank (Accession Numbers DQ167400.1). To increase the read coverage at the end of the CDS, their 5′ and 3′ ends were extended with 100-bp flanking regions, which were discarded after read mapping. These reads were obtained from RNA-seq data in the SRA database at NCBI (SRA accessions ERR748786 and ERR748787). Based on these two inputs, editing sites were identified by counting the number of A, C, G, and T nucleotides in reads aligned at each position of the reference ORFs. The editing level, T/(A+C+G+T), was calculated at each C-containing reference position. An editing site was defined when a C-containing reference position showed an editing level equal to or greater than 10%. The cut-off 10% was selected because sites with editing level less than 10% are often due to sequencing errors and are usually not conserved across different plant species as revealed previously [12,35,56].

To reduce the number of false positives, only paired-end and unique reads were selected, which increased the specificity of the read mapping. RNA-seq files were downloaded into a local computer to generate the SAM files using Magic-BLAST v 1.5.0 (https://ncbi.github.io/magicblast). Penalty of −4, gap open of 50, and gap extend of 50 were set to avoid partially edited transcripts and guarantee mapping reads from mature mRNAs. SAMtools v0.1.18 (https://github.com/genom e/bam-readcount) was used to filter trustless mapped reads, duplicated and unpaired reads. Two SAM files were merged together into a single SAM file with Picard tools v2.9.0 (http://broad institute.github.io/picard). Then, the merged SAM file was converted into a BAM file and sorted with SAMtools. With a sorted BAM file, bam-read count v0.8.0 was employed to count the number of A, C, G, and T nucleotides at each position.

### 4.4. Statistical Analysis

Correlation between edited Cs and total Cs or gene length was analyzed by Pearson’s correlation using the SPSS 22.0 software package.

## Figures and Tables

**Table 1:** Number of editing sites in rice mitochondria-encoded open reading frames (ORFs) reported by different studies.

Gene	Number of C-to-U Editing Sites		
	REDIdb	(Notsu et al., 2002)	In This Study
*atp1*	5	5	5
*atp6*	16	17	18
*atp9*	8	8	8
*ccmB* (*ccb2*)	35	35	35
*ccmC* (*ccb3*)	35	36	35
*ccmFC* (*ccb6c*)	27	27	27
*ccmFN* (*ccb6n*)	31	31	38
*cob* (*cytb*)	19	19	19
*cox1*	4	4	5
*cox2*	19	19	21
*cox3*	1	1	13
*matR*	0	0	16
*nad1*	23	23	26
*nad2*	30	30	32
*nad3*	15	15	19
*nad4*	20	20	21
*nad4L*	10	10	11
*nad5*	11	11	13
*nad6*	18	18	18
*nad7*	32	32	34
*nad9*	12	12	13
*orf25* (*atp4*)	9	9	9
*orf288*	0	0	3
*orfB* (*atp8*)	4	4	7
*orfX*(*mttb*)	33	33	37
*rpl2*	1	1	1
*rpl5*	1	1	1
*rpl16*	12	12	13
*rps1*	3	3	3
*rps2*	10	10	10
*rps3*	10	10	14
*rps4*	15	15	18
*rps7*	2	2	2
pseudo-*rps11*	4	4	5
*rps12*	0	0	6
*rps13*	8	8	7
*rps19*	6	6	6
*Total*	489	491	569

## Supplementary Materials

The following are available online at https://www.mdpi.com/2223-7747/9/10/1277/s1. Figure S1. Two editing sites detected by Sanger sequencing while undetected by RNA-seq. Figure S2. Five novel RNA editing sites in rice mitochondria-encoded *matR*. Table S1. Annotations of C-to-U RNA editing in rice mitochondria-encoded ORFs. Table S2. Identification of C-to-U RNA editing in the CDS of rice mitochondria-encoded ORFs by RNA-seq. Table S3. Extent of RNA editing in rice mitochondria-encoded ORFs. Table S4. Primers used in this study.
