# Supplementary material for: Detection and Analysis of C-to-U RNA Editing in Rice Mitochondria-Encoded ORFs
Source: Plants (Basel). 2020 Sep 28;9(10):1277. doi: 10.3390/plants9101277 (PMC7600565; doi:10.3390/plants9101277)
Supplement: Supplementary file 1 [file plants-09-01277-s001.zip › Figure S1-2.docx]

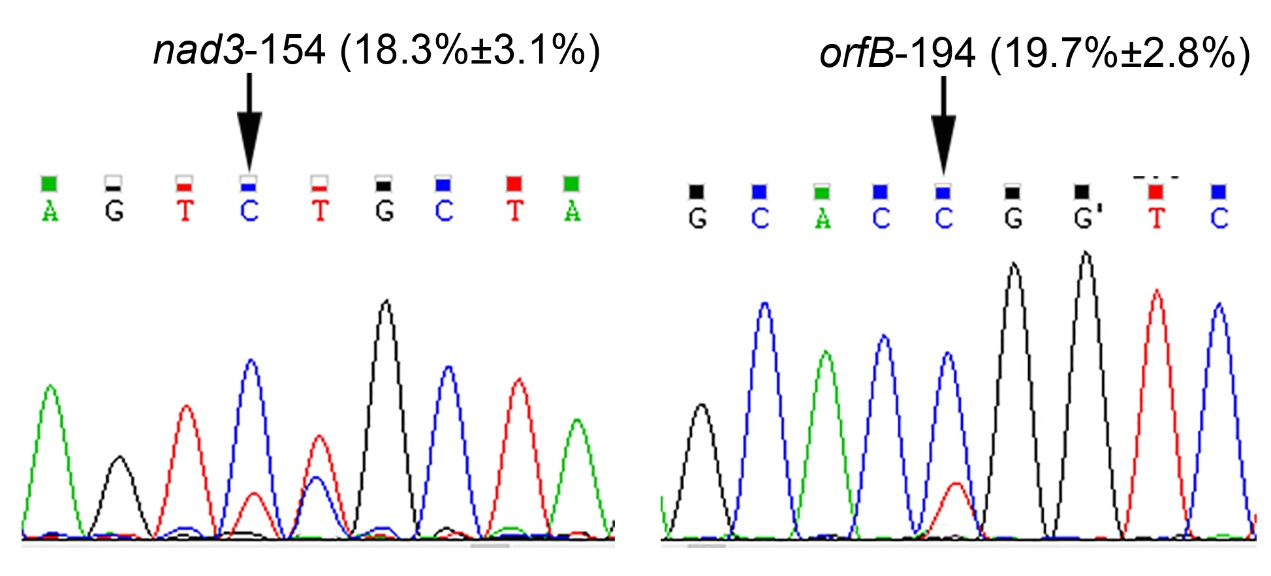


**Figure S1. Two editing sites detected by Sanger sequencing while undetected by RNA-seq.** Arrows above sequence chromatograms indicate the positions of editing sites *nad3-*154 and *orfB-*194. Editing levels C/(C+T) in parentheses were calculated based on the relative height of C and T.


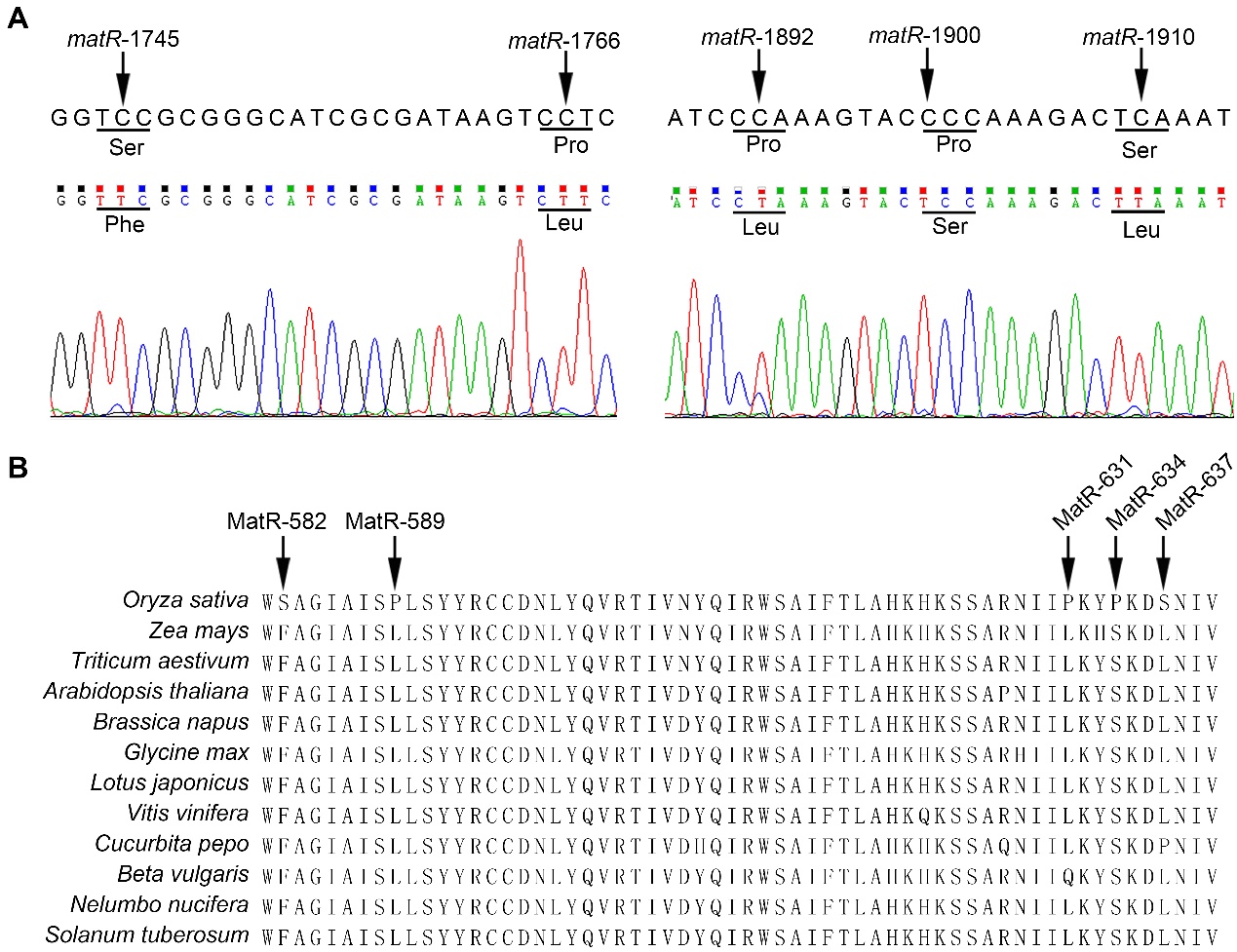


**Figure S2. Five novel RNA editing sites in rice mitochondria-encoded *matR*.** A, Sequence chromatogram of PCR-amplified *matR* cDNA. Arrow indicates the novel editing sites *matR-*1745, *matR-*1766, *matR-*1892, *matR-*1900, and *matR-*1910. Amino acids before and after editing are shown below the codons indicated by black lines. B, Alignment of amino acid sequences from rice MatR and its orthologs from other plant species. The amino acid sequence of *Oryza sativa* MatR was encoded by unedited CDS sequence, whereas its counterparts in *Zea mays, Triticum aestivum, Arabidopsis thaliana, Brassica napus, Glycine max, Lotus japonicus, Vitis vinifera, Cucurbita pepo, Beta vulgaris, Nelumbo nucifera,* and *Solanum tuberosum* were retrived from REDIdb. All the sequences were aligned with Clustal Omega (http://www.ebi.ac.uk/Tools/msa/clustalo/). Arrows indicate the amino acids for rice MatR-582, MatR-589, MatR-631, MatR-634, and MatR-637. Note that amino acid sequences from REDIdb are translated from edited transcripts.
